# Is It Really Third Ventricle? A Pitfall in the Diagnosis of Hydrocephalus by Brain Ultrasound

**DOI:** 10.1007/s12028-020-01060-9

**Published:** 2020-08-20

**Authors:** Anselmo Caricato, Eleonora Ioannoni, Camilla Gelormini

**Affiliations:** 1 Department of Anesthesiology and Intensive Care, Catholic University School of Medicine, Rome, Italy; 2 IRCCS Fondazione Policlinico Universitario “A. Gemelli”, UOS “Terapia Intensiva Neurochirurgica”, Largo A. Gemelli, 8 00168 Rome, Italy

A 32-year-old female was admitted in Neuro Intensive Care of Policlinico “A. Gemelli” in Rome after bleeding from artero-venous malformation and ventriculostomy. After 6 days, the patient was awake (Glasgow Coma Scale (GCS) 14;E4,V4,M6), and external ventricular drainage was removed. Two days after, she deteriorated (GCS 11;E3,V3,M5); a brain sonography was performed by a neuro-intensive care unit (ICU) resident, and the exam was considered normal. According to our policy for residents training, echography was immediately repeated by an expert neurosonologist (Anselmo Caricato), and in a mesencephalic plane, two central pulsating hyperechoic lines were observed (Fig. 1a). They were wrongly considered as third ventricle. Actually, in a diencephalic plane, third ventricle was dilated, and in a ventricular plane, frontal horns of lateral ventricles were enlarged (Fig. 1b, ESM). A CT scan was obtained (Fig. 1c), and ventriculostomy was immediately performed.

**Fig. 1:**
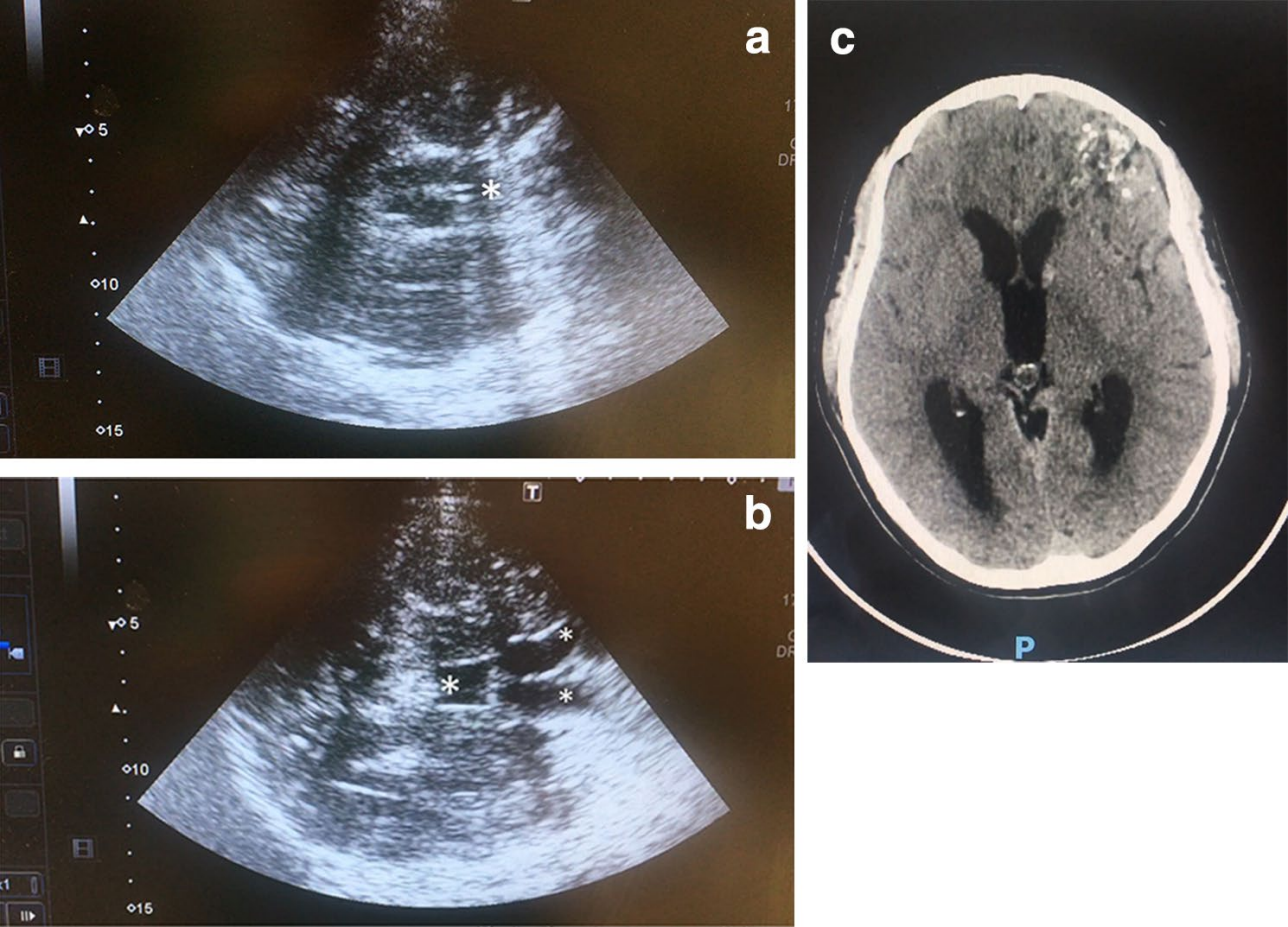
In a mesencephalic plane (**a**), two central pulsating hyperechoic lines, cerebral aqueduct (asterisk), were wrongly considered as third ventricle. Tilting the probe 10° cranially, in a diencephalic plane (**b**), third ventricle(large asterisk) and frontal horns of lateral ventricles (small asterisks) were observed, and hydrocephalus was diagnosed. A CT scan (**c**, EMS) confirmed the diagnosis

Brain sonography is a non-invasive monitoring method, and clinical applications have been described in several settings: it may be useful for bedside hematoma assessment, for midline shift evaluation, for diagnosis of hydrocephalus [1–4]. In particular, a good correlation has been observed between sonography and CT scan measurements of the width of the third ventricle, right and left frontal horns [5]. Furthermore, transcranial Doppler is recommended for vasospasm assessment after subarachnoid hemorrhage, and may be used for a non-invasive estimation of intracranial pressure [6, 7]. A recent review focused on indications and methods of this technique [8].

In our opinion, it is an excellent method for bedside diagnosis of hydrocephalus. It has a strong potential in neurointensive care, where early diagnosis is crucial, and transport to CT scan may be difficult and potentially dangerous. Ultrasound temporal window is generally good; Seidel was not able to visualize third ventricle for acoustic limitations in 7% of cases [5].

Actually, diagnosis of hydrocephalus requires expertise and hands-on training. In particular, cerebral ventricles can be often localized only by identification of ependyma, as two parallel pulsating hyperechoic lines, and not by visualization of cerebral spinal fluid; unfortunately, parallel hyperechoic lines are frequently observed in cerebral parenchyma, and can be incorrectly interpreted. If we exclude emergency procedures, we always recommend brain CT to confirm ultrasound data before ventriculostomy.

A comprehensive knowledge and a rigorous application of the method are of paramount importance for a correct identification of cerebral ventricles. By transtemporal window, brain ultrasound should be first performed identifying mesencephalic structures in an axial plane; in this view, basal cerebral arteries can be visualized, and color mode can be a simple tool helping in plane identification. Then, the probe should be tilted 10° cranially in a diencephalic plane, and third ventricle can be observed. This creates an error in the measurement of the transverse diameter of the ventricle, which, however, is generally acceptable; a further cranial inclination allows the identification of the lateral ventricles and of the thalamus, but generates an error in the measurement of the transverse diameter of the lateral ventricle which may not be negligible. This should be considered, in particular if we want to obtain a measurement of transverse diameter of frontal horn of contralateral ventricle. A complete exam requires the study of mesencephalic, diencephalic and ventricular plane.

In our case, the mistake was caused by an incorrect identification of insonation planes, and by a not complete execution of the exam. In fact, third ventricle cannot be observed in a mesencephalic plane, and two parallel pulsating hyperechoic lines corresponded to a dilated cerebral aqueduct, and not to third ventricle. In patients without hydrocephalus, cerebral aqueduct is generally not visible by ultrasound. By simply tilting the probe 10° cranially, the dilated third ventricle could be observed.

In conclusion, hydrocephalus can be bedside observed by brain ultrasound, but requires expertise. By using a transtemporal window, a correct identification of plane of insonation is mandatory. It has always to be performed according a systematic approach that includes a complete examination of mesencephalic, diencephalic and ventricular plane.

So far, unlike in other fields such as echocardiography, certification of competences in brain ultrasound by intensive care medicine societies is lacking [9]. In our University hospital, brain ultrasound is included in critical care training program for residents; we believe that it should not be considered only as a useful adjunct, but a standard competence of critical care specialist.

## Electronic supplementary material

Below is the link to the electronic supplementary material.

Supplementary file1 (MOV 22677 kb)
